# Extending Treatment Networks in Health Technology Assessment: How Far Should We Go?

**DOI:** 10.1016/j.jval.2015.03.1792

**Published:** 2015-07

**Authors:** Deborah M. Caldwell, Sofia Dias, Nicky J. Welton

**Affiliations:** School of Social and Community Medicine, University of Bristol, Bristol, UK

**Keywords:** comparative effectiveness, health technology assessment, literature searching, mixed treatment comparisons, network meta-analysis, systematic review

## Abstract

**Background:**

Network meta-analysis may require substantially more resources than does a standard systematic review. One frequently asked question is “how far should I extend the network and which treatments should I include?”

**Objective:**

To explore the increase in precision from including additional evidence.

**Methods:**

We assessed the benefit of extending treatment networks in terms of precision of effect estimates and examined how this depends on network structure and relative strength of additional evidence. We introduced a “star”-shaped network. Network complexity is increased by adding more evidence connecting treatments under five evidence scenarios. We also examined the impact of heterogeneity and absence of evidence facilitating a “first-order” indirect comparison.

**Results:**

In all scenarios, extending the network increased the precision of the A versus B treatment effect. Under a fixed-effect model, the increase in precision was modest when the existing direct A versus B evidence was already strong and was substantial when the direct evidence was weak. Under a random-effects model, the gain in precision was lower when heterogeneity was high. When evidence is available for all “first-order” indirect comparisons, including second-order evidence has limited benefit for the precision of the A versus B estimate. This is interpreted as a “ceiling effect.”

**Conclusions:**

Including additional evidence increases the precision of a “focal” treatment comparison of interest. Once the comparison of interest is connected to all others via “first-order” indirect evidence, there is no additional benefit in including higher order comparisons. This conclusion is generalizable to any number of treatment comparisons, which would then all be considered “focal.” The increase in precision is modest when direct evidence is already strong, or there is a high degree of heterogeneity.

## Introduction

Indirect comparisons and network meta-analysis (NMA) are increasingly common in the evaluation of multiple competing health technologies when interest lies in the relative rankings of all treatments of clinical interest [1]. NMA is also used by health reimbursement agencies worldwide, including the National Institute for Health and Care Excellence (NICE) Single Technology Appraisals (STAs) program, where the objective is to assess whether a treatment should be available for use on the National Health Service in England and Wales. STAs are the mainstay of the NICE health technology assessment (HTA) program; of the 33 appraisals published in 2013, 29 were completed under the STA process (www.nice.org.uk). STAs typically evaluate a single treatment close to marketing launch, and as such the focal comparison of interest is with standard/usual care options. We note that this is true even when multiple treatments are included in a network and relative rankings reported [2].

NMA may be used in STAs when direct evidence from trials of A versus B is either unavailable or sparse; however, no formal guidelines exist to ensure transparency on which treatments should be included, when to extend a network, or how far it should be extended. In the absence of such guidelines, there are concerns that networks could be defined specifically to favor a particular treatment [3,4]. Proposals for the assessment of network geometry have received attention [5,6], and network size has been described as an “unsolved issue” in NMA [7]. In an empirical study of 18 published networks, Mills et al. [8] examined the impact of retrospectively excluding treatments and note how treatment effect estimates and treatment rankings were modified. In STAs, however, the starting network consists of a *fixed* “decision set” of treatments (i.e., treatment and comparator(s) of interest) to which additional evidence (a “supplementary set” of treatments identified a priori) may be prospectively included to connect those already in the network. Such an approach has been separately described by Ades et al. [9] and Hawkins et al. [10] and is referenced by ISPOR Task Force [11] and NICE methodology guidelines [12].

A recent case study calls for further work to evaluate network size and structure and provide generalizable findings on the added value of extending treatment networks [13]. Indeed, there is a practical need to ask how far to extend a network in STAs [14], what is the benefit of doing so, and whether there is a diminishing return for including additional treatments. NMA is understood to be more resource intensive than traditional pairwise systematic review [15]. For example, literature searching, screening, eligibility assessment, and data extraction may be more cumbersome because of the increased number of studies to review, although this will vary depending on the network. The further a network is extended, the risk of bias, heterogeneity, and inconsistency may also increase. This would further add to the reviewer’s workload assessing whether the assumption of consistency/transitivity holds across the network [16]. However, previous empirical work suggests that combining direct and indirect evidence may increase the precision of treatment effect estimates across a network [17]. Taking the perspective that the purpose of evidence synthesis is to reduce uncertainty in decision making, a key consideration in the development of guidelines on how far to extend evidence networks is the impact on the precision of the focal treatment comparison(s).

In this article, we explore the effect of combining direct and indirect evidence in an NMA on the precision of a single pairwise comparison in a hypothetical six-treatment network. Our starting point is to assume that a literature search has been conducted and has generated a “star”-shaped starting network. We explore the effects of “extending” the network by including additional evidence situated at different points in the network. The article is structured as follows. First, we define the statistical properties of indirect comparisons. Then, we introduce the network structure and describe the different evidence scenarios considered here. The statistical method is described and findings are reported. We conclude by discussing the practical implications of the findings, make recommendations for the systematic review component of HTA, and discuss implications for NMA, in general.

## Methods

In a three-treatment network, an indirect estimate of the A versus B treatment effect estimate is derived as follows:

(1) $$\theta_{\mathrm{AB}}^{I}\;=\;\theta_{\mathrm{AC}}^{D}\;-\;\theta_{\mathrm{BC}}^{D}$$

where θ represents a treatment effect estimate (e.g., log-odds ratio, mean difference) and where superscript I denotes an indirect estimate and superscript D denotes a direct estimate. The variance of $\theta_{\mathrm{AB}}^{I}$ is equal to the sum of the variances ${\hat{V}}_{\mathrm{AC}}^{D}\;and\;{\hat{V}}_{\mathrm{BC}}^{D}$ estimated from the direct A versus C and B versus C comparisons, ${\hat{V}}_{\mathrm{AB}}^{I}={\hat{V}}_{\mathrm{AC}}^{D}+{\hat{V}}_{\mathrm{BC}}^{D}$. Here, we define A and B as our focal treatments of interest. Any comparison of A or B to another treatment (e.g., C) is defined as contributing “first-order” evidence if it facilitates a triangular loop (e.g., A vs. C and B vs. C) [10]. A comparison that does not include either A or B but that facilitates a quadrilateral loop of evidence (e.g., C vs. D in the loop A-B-C-D) is defined as providing “second-order” evidence for the focal treatments of interest A and B.

### Network Formation

**Fig. 1:**
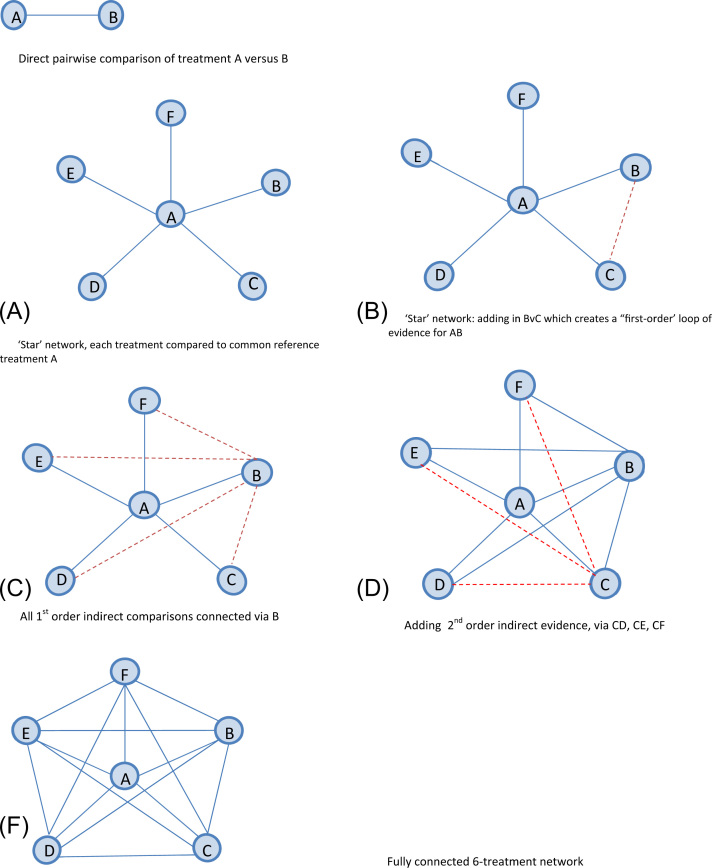
Graphical representation of the star network and approach to connecting the network under the assumption of all available evidence. The solid blue line indicates existing evidence. The dotted red line indicates evidence added at each step of extension. (**A**) Direct pairwise comparison of treatment A versus B. (B) “Star” network, each treatment compared with common reference treatment A. (C) “Star” network: adding in B vs. C, which creates a “first-order” loop of evidence for AB. (D) All first-order indirect comparisons connected via B. (E) Adding second-order indirect evidence, via CD, CE, and CF. (F) Fully connected six-treatment network.

Our starting point was to assume that a literature search has been conducted and has generated a network with six treatments labeled A, B, …, F, where treatments A and B form the “decision set” of treatments and the effect estimate of interest is $\theta_{\mathrm{AB}}$. For simplicity, we assume a known network size, such that all possible comparisons can be known a priori. Six is the median number of treatments observed in published NMAs [18]. In a standard systematic review, only direct evidence on contrast A versus B (Fig. 1A) would be reported, which represents a single pairwise meta-analysis here. Note that the solid lines connecting each pair of treatments in Figure 1 indicate that there is direct evidence available for that contrast. Drawing on the principles of an iterative strategy for NMA [10], we assume that evidence “closest” to the focal treatment comparison of interest will be included first. Here we first add evidence on all comparisons including treatment A, forming a “star” network structure (Fig. 1B). We then add evidence that forms triangular “first-order” loops for A versus B (B vs. C, B vs. D, B vs. E, and B vs. F) [19] (Fig. 1C,D). Second-order indirect evidence, via treatment C, is added next (Fig. 1E). The final level of network complexity (Fig. 1F) is to include all evidence via D versus E, D versus F, and E versus F.

### Description of Evidence Scenarios

#### (i) Network with Evidence Available for All Contrasts

Here we concentrate on a network structure in which direct evidence *is* available for $\theta_{\mathrm{AB}}$, albeit in differing amounts. Five *hypothetical* scenarios are considered under an assumption of consistency (Equation 1). In each scenario, we assume that values for the observed precision of treatment effect estimates are available for every pairwise contrast. The resulting precision of the pooled NMA estimate for A versus B depends only on these input precisions and not on the actual observed treatment effects (see Appendix 1 in Supplemental Materials found at doi:10.1016/j.jval.2015.03.1792). No assumptions are made about the observed treatment effects, and results are general for any outcome measure with our assumed input precisions. Furthermore, our conclusions are based on the relative precision across different parts of the network, rather than on the absolute value. Input precision values for each scenario are reported in Table 1.

***Scenario 1:** Equal variance is assumed for each contrast across the network*. Here, each contrast $\theta_{\mathrm{XY}}$ is informed by a meta-analysis with variance, *V*_*XY*_ *=* 1, where *V*_*XY*_ is the observed variance (SE^2^) from a meta-analysis of *X* versus *Y*. The precision of *X* versus *Y* is defined as $P_{\mathrm{XY}}^{D}=1/V_{XY}$.

**Scenario 2:** A versus B comparison is the “weakest” link in the six-treatment network. Contrasts contributing first-order indirect evidence are also weak (imprecise), and second-order contrasts contribute even weaker evidence for A versus B. This scenario is sometimes seen when fewer trials are conducted for ethical or practical reasons, for example, in pain management for women in labor [20]. Note that values assigned in all scenarios are hypothetical, and do not exactly replicate the illustrative HTAs.

**Scenario 3:** The A versus B comparison is the “weakest” link in the six-treatment network, with the contrasts forming both first- and second-order indirect comparisons being stronger. In HTA, this scenario is seen when A versus B are interventions from rival manufacturers that have seldom been compared, or are compared only in a small study [21]. Evidence in such networks is likely to be found on the newer technologies versus placebo/standard care and on the standard versus older interventions.

**Scenario 4:** A versus B is the strongest link in the six-treatment network, with the contrasts forming indirect comparisons being weaker. This scenario may be seen in practice when both A and B are older interventions, perhaps the criterion standards for the clinical area, and have been trialed many times [22].

**Scenario 5:** A versus B comparison is the strongest link, with the contrasts contributing to indirect comparisons also being strong. This scenario may be seen in practice with “me-too” pharmaceutical treatments such as selective serotonin reuptake inhibitors [23] and treatments for hyperphosphatamia [24].

#### *(ii)Absence of first-order indirect evidence for*$\theta_{\mathrm{AB}}$

**Fig. 2:**
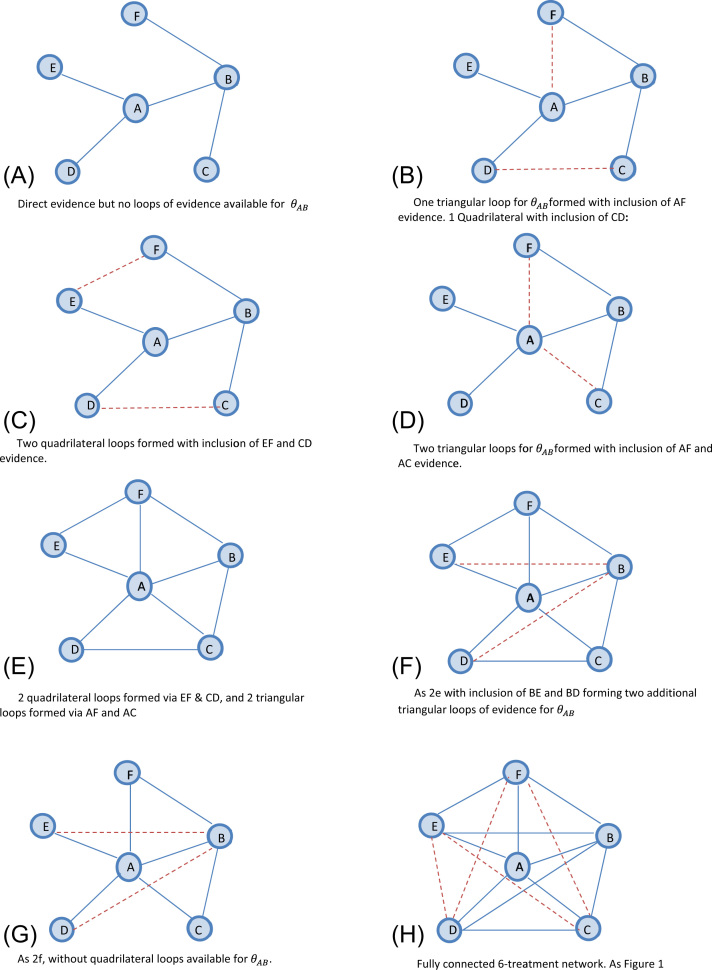
Graphical representation of network and approach to connecting the network under assumption of missing first-order indirect evidence. The solid blue line indicates existing evidence. The dotted red line indicates evidence added at each step of extension. (A) Direct evidence but no loops of evidence available for *θ*_*AB*_. (B) One triangular loop for *θ*_*AB*_ formed with the inclusion of AF evidence. One quadrilateral with the inclusion of CD. (C) Two quadrilateral loops formed with the inclusion of EF and CD evidence. (D) Two triangular loops for*θ*_*AB*_ formed with the inclusion of AF and AC evidence. (E) Two quadrilateral loops formed via EF and CD, and two triangular loops formed via AF and AC. (F) As 2E with the inclusion of BE and BD forming two additional triangular loops of evidence for *θ*_*AB*_. (G) As 2F, without quadrilateral loops available for *θ*_*AB*_. (H) Fully connected six-treatment network. As Figure 1.

In (i) above, we assumed that evidence is available for every contrast in the six-treatment network. This, however, may not be the case in practice. Here, we also explore the impact of absent first-order evidence for $\theta_{\mathrm{AB}}$. The network is extended as follows and with reference to Figure 2: We start with a network in which evidence is available on five edges of the network, A versus B, A versus D, A versus E, B versus C, and B versus F (Fig. 2A), but no loops—triangular or quadrilateral—are available. In Figure 2B, we complete a single first-order loop via A versus F and a single second-order loop via C versus D. In Figure 2C, there are two quadrilateral loops formed by adding E versus F and C versus D evidence to the network. This allows second-order indirect comparisons for $\theta_{\mathrm{AB}}$. This is compared with Figure 2D, which instead includes A versus C and A versus F evidence to complete two triangular loops for $\theta_{\mathrm{AB}}$. In Figure 2E, we include two quadrilateral and two triangular loops (i.e., Fig. 2B,C combined). Alternative permutations of evidence unavailability are considered in Figure 2F,G. Figure 2F considers a network with four triangular and two quadrilateral loops for $\theta_{\mathrm{AB}}$. This is compared with Figure 2G, in which the quadrilateral loops are unavailable. For completeness, Figure 2H reports a fully connected network; however, it is identical to that in Figure 1F. The same hypothetical scenarios are considered as for (i) and the same values used for the variances as reported in Table 1.

### Statistical Analysis

An algebraic solution for the posterior precision $P_{\mathrm{AB}}^{\mathrm{NMA}}$ for $\theta_{\mathrm{AB}}$ for a given scenario (network structure and input precisions) can be written down for both fixed- and random-effects models (see Appendix 1 in Supplemental Materials). This allows us to explore this mathematical relationship without the need for a simulation study. Appendix 1 shows how the posterior precision $P_{\mathrm{AB}}^{\mathrm{NMA}}$ can be computed for the fixed- and random-effects models and provides further technical details for the analysis.

Under each of Scenarios 1 to 5 outlined above, $P_{\mathrm{AB}}^{\mathrm{NMA}}$ is computed for every level of “connectednesses” of the network (Fig. 1). The increase in precision from the NMA over the direct evidence, $\Delta =P_{\mathrm{AB}}^{\mathrm{NMA}}-P_{\mathrm{AB}}^{D}$, is calculated and reexpressed as a percentage increase. For the random-effects models, we explored the effect of differing degrees of between-study heterogeneity variances, τ^2^. We based our choice of τ^2^ = 0.1, 0.5, and 1 (on the log-odds scale) on findings from a meta-epidemiological database of 234 meta-analyses that provides a range of τ = 0 to 1.33 on the log-odds scale [25]. We might expect a similar range of values on the standardized mean difference scale too, and the qualitative, if not quantitative, results to apply on other outcome measures. Appendix Table 1 in Supplemental Materials found at doi:10.1016/j.jval.2015.03.1792 provides further details on the between-trial variance.

## Results

**Fig. 3:**
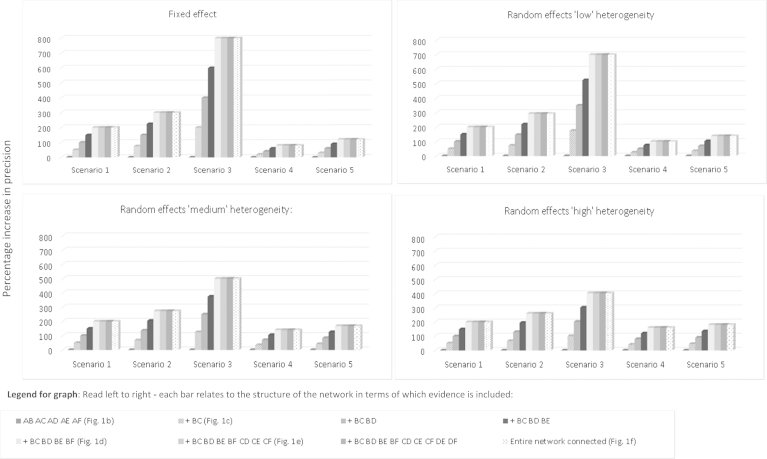
Star network with six treatments for all available evidence. Comparison of percentage increase in precision (y-axis) for treatment contrast A vs. B from expanding the network of treatments under five separate scenarios over that achieved from a standard, pairwise meta-analysis. Scenarios considered under fixed- and random-effects models. Random-effects models assume “low,” “medium,” and “high” levels of heterogeneity as defined in the main text. The height of each bar denotes the percentage increase in the precision of A vs. B treatment effect estimate from a network meta-analysis (NMA). The horizontal axis reports each increasing level of the network under each of the five scenarios. Read left to right**—**Each bar relates to the structure of the network in terms of which evidence is included. **Scenario 1:** “One trial per comparison”: Equal variance across the network. Each comparison XY represents one meta-analysis with variance equal to 1. **Scenario 2:** “AB weakest link, IC trials weaker”: AB comparison is the “weakest” link, with the comparisons forming ICs being weaker. **Scenario 3:** “AB weakest link, IC trials strong”: AB comparison is the “weakest” link, with the comparisons forming ICs being stronger. **Scenario 4:** “AB strongest link, IC trials weaker”: AB comparison is the “strongest” link, with the comparisons forming ICs being weaker. **Scenario 5:** “AB strongest link, IC trials strong”: AB comparison is the “strongest” link, with the comparisons forming ICs also being strong. IC, indirect comparisons.

**Fig. 4:**
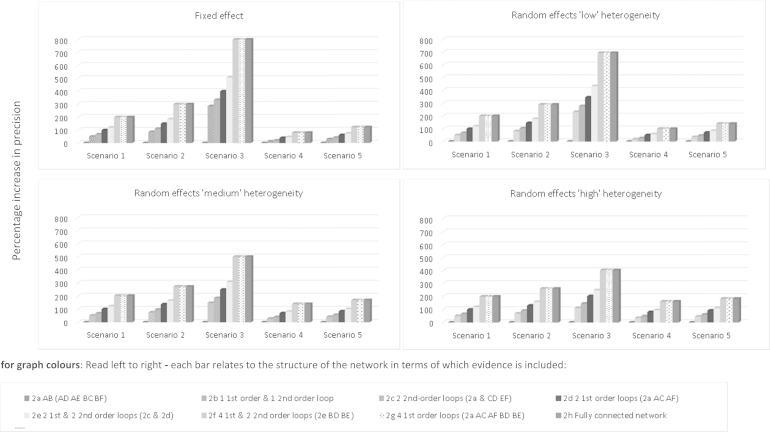
Star network with six treatments when first-order indirect evidence is unavailable. Comparison of percentage increase in precision (y-axis) for treatment contrast A vs. B from expanding network of treatments under five separate scenarios over that achieved from a standard, pairwise meta-analysis. Scenarios considered under fixed- and random-effects models. Random-effects models assume “low,” “medium,” and “high” levels of heterogeneity as defined in the main text. The height of each bar denotes the percentage increase in precision of A vs. B treatment effect estimate from a network meta-analysis (NMA). The horizontal axis reports each increasing level of the network under each of the five scenarios. For graph colors: Read left to right**—**Each bar relates to the structure of the network in terms of which evidence is included. **Scenario 1:** “One trial per comparison”: Equal variance across the network. Each comparison XY represents one meta-analysis with variance equal to 1. **Scenario 2:** “AB weakest link, IC trials weaker”: AB comparison is the “weakest” link, with the comparisons forming ICs being weaker. **Scenario 3:** “AB weakest link, IC trials strong”: AB comparison is the “weakest” link, with the comparisons forming ICs being stronger. **Scenario 4:** “AB strongest link, IC trials weaker”: AB comparison is the “strongest” link, with the comparisons forming ICs being weaker. **Scenario 5:** “AB strongest link, IC trials strong”: AB comparison is the “strongest” link, with the comparisons forming ICs also being strong. IC, indirect comparision.

Figures 3 and 4 report findings for both network structures and every evidence scenario. Results for each network structure are reported separately.

### (i)Network Structure with Evidence Available for All Contrasts

Figure 3 reports the findings from the network structure in which we assume evidence is available for all contrasts. The categories on the horizontal axis correspond to the five evidence scenarios outlined above. Within each scenario, different levels of network connectedness are considered as represented in the network diagrams shown in Figure 1. For example, the first category corresponds to Figure 1A in which only direct evidence is available for A versus B, and the second category corresponds to Figure 1C, in which the B versus C evidence is added to the network. The remaining categories introduce the treatment comparisons in order of increasing complexity of the evidence network. The vertical axis plots the difference in precision, Δ, as a percentage increase. See also Appendix Tables 2 to 5 in Supplemental Materials found at doi:10.1016/j.jval.2015.03.1792. Across all five scenarios, $P_{\mathrm{AB}}^{D}$ is obtained solely from the direct A versus B evidence and therefore is always equal to the observed pairwise precision as defined in the individual scenarios. At the first level of the NMA (corresponding to Fig. 1B), $P_{\mathrm{AB}}^{\mathrm{NMA}}$ is also equivalent to information gained solely from direct A versus B meta-analysis and therefore Δ = 0 (0% increase in precision).

The results from the fixed-effect analyses are shown in the top left-hand panel of Figure 3. Having added first-order indirect evidence on B versus C to the network, a triangular loop A-B-C (Fig. 1C) is formed, thereby allowing both an indirect and a direct estimate of ${\hat{\theta }}_{\mathrm{AB}}$. Under Scenario 1, $P_{\mathrm{AB}}^{\mathrm{NMA}}$ is increased to 1.50, with Δ = 0.50 (50% increase). For this scenario only, this increase can be interpreted as equivalent to additional information gained from a trial of half the size of the “direct” A versus B study. As network complexity increases, we note that evidence on each additional first-order comparison increases $P_{\mathrm{AB}}^{\mathrm{NMA}}$ by 0.5. For example, having added first-order evidence B versus C and B versus D, we see $P_{\mathrm{AB}}^{\mathrm{NMA}}$ has increased to 2.00 (Δ = 1.00 or 100% increase). This can be interpreted as having increased the precision by the equivalent of one additional randomized controlled trial “worth” of information. When evidence on B versus E and B versus F is added to the network (Fig. 1E), however, we note a “ceiling effect” after which including further evidence does not increase $P_{\mathrm{AB}}^{\mathrm{NMA}}$.

Figure 3 also reports the results from four further scenarios for the “star” network under the fixed-effect model. When the A versus B comparison has the largest variance (i.e., is the weakest link in the network), the additional benefit of including indirect evidence is substantial (Scenario 3). For example, including evidence on all first-order indirect comparisons results in an 800% increase in $P_{\mathrm{AB}}^{\mathrm{NMA}}$. Conversely, under Scenario 4, when the A versus B comparison already has the greatest amount of information, including further evidence to facilitate an indirect comparison results only in a modest increase in the A versus B precision. For example, including evidence on all first-order indirect comparisons (B vs. C, ..., B vs. F) results in an 80% increase in $P_{\mathrm{AB}}^{\mathrm{NMA}}$. A ceiling effect is again evident across all scenarios. Once all evidence facilitating a first-order indirect comparison has been included in the network, there is no further increase in precision gained from including the second-order indirect evidence for $\theta_{\mathrm{AB}}$.

Figure 3 also reports the findings under an assumption of random treatment effects, and varying amounts of heterogeneity. Across all levels of heterogeneity, the observed pattern is similar to that seen under a fixed-effect assumption and we again note a ceiling effect. The largest increase in precision is seen when the A versus B evidence is uncertain and the evidence contributing to indirect comparisons is strong (Scenario 3), and the smallest increase is observed under Scenario 4 when the A versus B evidence is already precise. Across all scenarios we note that the absolute increase in $P_{\mathrm{AB}}^{\mathrm{NMA}}$ is greatest when τ^2^ = 0.1 and is least when τ^2^ = 1. If one considers the most conservative scenario under considerable heterogeneity (Scenario 4, where τ^2^ = 1), the increase in $P_{\mathrm{AB}}^{\mathrm{NMA}}$ is still a substantial 161% (see Appendix Table 4 in Supplemental Materials). Comparing the percentage increase in precision, we note that smaller relative gains in precision are observed between each level of network connectedness when direct evidence is weak (Scenarios 2 and 3) and heterogeneity is “large” (τ^2^ = 1) than when τ^2^ = 0.1. When direct evidence is strong (Scenarios 4 and 5), the reverse is observed.

For completeness, Appendix 2 in Supplemental Materials found at doi:10.1016/j.jval.2015.03.1792 reports the findings from a series of sensitivity analyses to investigate the impact of both increasing the size of the variance inputs and varying them within the level of evidence for the six-treatment network. Although the increase in $P_{\mathrm{AB}}^{\mathrm{NMA}}$ is dependent on the strength of the data inputs, we note that the observed “ceiling effect” remains evident when all first-order evidence has been included.

### *(ii)Absence of First-Order Indirect Evidence for*$\theta_{\mathrm{AB}}$

Figure 4 reports the findings from the network when we assume that indirect evidence is unavailable (for some contrasts) under the five evidence scenarios. Results are reported with reference to Figure 2. Numerical results are reported in Appendix 1 in Supplemental Materials (see Appendix Tables 6–9 in Supplemental Materials found at doi:10.1016/j.jval.2015.03.1792).

Focusing on the fixed-effect model, under Scenario 1, in the absence of evidence facilitating a first-order indirect comparison, there is a small benefit achieved by including second-order evidence; $P_{\mathrm{AB}}^{\mathrm{NMA}}$ is increased to 1.33, with Δ = 0.33 (33% increase). For Figure 2C, in which there are two second-order indirect comparisons available, we observe $P_{\mathrm{AB}}^{\mathrm{NMA}}$ = 1.67, with Δ = 0.67 (67% increase). In Figure 2B, in which only one first-order indirect comparison and one second-order comparison are available (Fig. 2B), $P_{\mathrm{AB}}^{\mathrm{NMA}}$ is increased to 1.83 (83% increase). Therefore, $P_{\mathrm{AB}}^{\mathrm{NMA}}$ has increased by 33% by including one second-order comparison; however, recall from Figure 1C that $P_{\mathrm{AB}}^{\mathrm{NMA}}$ increased by 50% if only one first-order comparison is included (Fig. 1C).

If two first-order comparisons are available (Fig. 2D), $P_{\mathrm{AB}}^{\mathrm{NMA}}$ is increased to 2.00, a 100% increase over that afforded from the direct evidence alone. We observe a marginal benefit of including second-order evidence in the presence of two first-order loops (Fig. 2E) as $P_{\mathrm{AB}}^{\mathrm{NMA}}$ is increased to 2.20.

This exploration again illustrates a “ceiling effect” as observed in the “all available” evidence structure—there is no additional increase in $P_{\mathrm{AB}}^{\mathrm{NMA}}$ once all first-order evidence has been included in the network. In Figure 2H, a fully connected network increases $P_{\mathrm{AB}}^{\mathrm{NMA}}$ to 3.00. If this is compared with Figure 2G, however, the removal of second-order evidence does not affect the precision gained. Figure 4 also reports the findings under an assumption of random treatment effects, and varying amounts of heterogeneity. Across all levels of heterogeneity, the observed pattern is similar to that seen under a fixed-effect assumption. For completeness, all numerical results are reported in Appendix 1 in Supplemental Materials (see Appendix Tables 6–9 in Supplemental Materials).

A sensitivity analysis was also conducted to investigate the impact of allowing information on the second-order evidence to outweigh that available for the direct and first-order evidence. Under a fixed-effect assumption, we note that it is only when the precision of the second-order evidence outweighs the first-order evidence by 400:1 that the precision gained by a single first-order loop is equivalent to the precision gained by a single second-order (quadrilateral) loop. We also note that the increase in precision achieved via both a first- and second-order loop of evidence is the same as that achieved by either two second-order loops or two first-order loops. Complete findings for the six-treatment network are reported in Appendix 3 in Supplemental Materials found at doi:10.1016/j.jval.2015.03.1792.

## Discussion

In this article, we explored the effects of extending a six-treatment network under two structures and five different evidence scenarios, in which the amount of information available for direct and indirect evidence was varied. Under both evidence structures, extending the network increased the precision of the treatment comparison of interest, $\theta_{\mathrm{AB}}$. This finding supports previous empirical investigations [17,26,27] and builds on those examining simpler evidence structures or single case studies [13,28,29]. We note that precision increased under all five scenarios, suggesting that regardless of the initial strength of the direct, first-order and second-order indirect evidence, combining them in an NMA increases the precision of $\theta_{\mathrm{AB}}$. For the structure for which all evidence was available, the percentage precision gained was most striking under a fixed-effect model in which the initial direct evidence was weak, the first-order indirect evidence was marginally stronger, and the second-order indirect evidence was strongest (Scenario 3). Conversely, the smallest increase (albeit still an increase of 80%) was observed under Scenario 4 when the $\theta_{\mathrm{AB}}$ direct evidence was already strong. These findings suggest that even when first-order indirect evidence is imprecise it should still be combined with direct evidence to increase the overall precision. Of interest, however, was the observation of a “ceiling effect” beyond which no further increase in precision was achieved by including comparisons contributing to a higher-order loop.

For the fixed-effect analyses assuming the absence of first-order indirect evidence, we observed that including second-order evidence affords a small increase in precision only *if* first-order indirect evidence is unavailable and even then this gain is minimal. This can be termed a “weakest link” effect, and similar findings have been observed in simulation studies [29]. In a sensitivity analysis, we observed that even when the precision of the second-order indirect evidence was 400 times greater than that of the first-order, the precision gained from the second-order loop did not exceed that gained from the first-order loop (should it be available).

Similar to Song et al. [28], we noted that the increase in precision was attenuated under extreme heterogeneity. Under all five scenarios and in both evidence structures, our findings were robust to increasing the number of studies per comparison in the random-effects analyses (holding overall precision constant). In a standard random-effects meta-analysis, a large heterogeneity variance typically leads to greater uncertainty in treatment effect estimates. Within an NMA, in which the heterogeneity parameter is shared (common) across all comparisons, this has generated a concern that the presence of extreme heterogeneity of treatment effects in an NMA may *de*crease the precision of effect estimates. We did not explore this possibility in our analysis because we assumed that the heterogeneity parameter was unchanged by the addition of extra trials. Here, we also assumed that the heterogeneity parameter is common across treatment comparisons. Relaxing this assumption by introducing a heterogeneous variance model [30] will change the strength of the evidence from each pairwise comparison in the network, and will therefore have an impact on precision of the focal A versus B comparison. We do not anticipate, however, that the general conclusions we draw from this article will change.

The observed “ceiling effect” occurred when all first-order evidence had been included in the network. This effect was evident under both fixed- and random-effects models, and was not dependent on the strength or position of information in the network. Although we used a different approach, this effect is consistent with König et al.’s [31] observation that networks can be reduced to first-order comparisons. In explaining the ceiling effect, we find it instructive to use the analogy of Ru¨cker [32] in which an NMA is likened to an electrical network. Under an assumption of equal variance, we observed that information in a network follows a “path of least resistance.” Where a shorter “path” to the comparison of interest exists in a network, including a treatment that facilitates an additional longer “path” did not increase $P_{\mathrm{AB}}^{\mathrm{NMA}}$. That is, in a network with both an A-B-C and an A-B-C-D loop of evidence, the greatest gain in $P_{\mathrm{AB}}^{\mathrm{NMA}}$ will be achieved via the shorter “path” A-B-C. Because the B versus C edge must also be used for the C versus D comparison to contribute to A versus B, the additional gain in $P_{\mathrm{AB}}^{\mathrm{NMA}}$ will be limited. This observation has important implications for the debate surrounding the inclusion of older treatments and placebo in NMAs [4] and crucially whether to include trials comparing one with the other. Certainly, any comparison of an older and placebo treatment will be second-order indirect evidence, and the results here suggest that they are unlikely to contribute to the precision of the effect estimate(s) of interest. Unless treatments are of direct interest to the decision maker, there may be a diminishing return for including second-order evidence comparing older versus placebo in an NMA.

Although a limitation of our approach is that we focused on the single comparison A versus B, we note that conclusions will generalize to networks in which multiple treatments are of interest. For example, if the focal treatments of interest are A, B, and C, then all first-order comparisons for A, all first-order comparisons for B, and all first-order comparisons for treatment C should be included. These conclusions have implications for the scoping and searching stages of a systematic review and are supportive of the iterative search strategy proposed by Hawkins et al. [10]. Although their proposal allows for any number of higher order indirect comparisons to be included, they speculate that reviewers may decide that it is not worthwhile to do so. Our results confirm that to maximize the cost-of-searching versus the value-of-the-evidence “trade-off,” systematic reviewers should include only those additional nonfocal treatments that have been compared with at *least two* of the focal treatments, that is, first-order evidence, to form a triangular loop in a network. Beyond this, our results show little merit, in terms of precision, in searching for further higher order evidence—except in the absence of first-order indirect evidence (although the gain in precision is small). For further discussion of the Hawkins approach, readers are referred to Dequen et al. [13] and Hawkins et al. [33].

Our objective in this article was to explore the increase in precision from including additional evidence. This does not depend on the observed effect estimates (see Appendix 1 in Supplemental Materials), and hence not on whether the additional evidence is inconsistent with the observed A versus B data. Nevertheless, the potential for increased bias/inconsistency as the network is extended further from the focal comparison(s) of interest is an important question to address. Previous work has examined the impact on treatment effect estimates and whether they are over- or underestimated in the presence of bias/inconsistency [34,35]. Our results suggest that first-order indirect evidence increases precision; therefore, if there is inconsistency between direct and indirect evidence, effect estimates will be influenced. We note, however, that including second-order indirect evidence adds little precision in the presence of the first-order evidence. As such, if there is inconsistency between the second-order indirect and direct evidence, we would expect the effect estimate to be driven by the direct first-order evidence, and not greatly influenced by the inconsistent second-order evidence. This is reassuring if we expect evidence further from the focal comparison to be at a higher risk of bias.

In the context of HTA, in which a cost-effectiveness model is informed by the NMA estimates, even small gains in precision may have implications for reimbursement decisions. This can be explored using value of information calculations [36], which measure the impact of increased precision in model inputs on a resulting reimbursement decision. If there is a high value in reducing uncertainty in the relative treatment effects, it may be worth initially extending the network to obtain an increase in precision before considering conducting further primary research. Similarly, if different point estimates are obtained from including different evidence networks, the resulting decision may change.

Of course, which treatments to include in an NMA should be primarily determined by the decision question. Treatments are not included in an NMA solely for the purpose of increasing the precision of effect estimates. Higher order indirect comparisons are likely to be included to link networks and estimate heterogeneity and meta-regression parameters. They may also facilitate treatment effect estimates when there is no head-to-head evidence and allow simultaneous comparison of all competing treatments, for example, by ranking them according to relative efficacy [37]. At present, there are no formal guidelines to ensure transparency on when to extend a network or how far it should be extended. Optimizing precision, while keeping networks manageable, is a principle that could be applied to develop such guidelines.

Source of financial support: D.M.C. was funded by an MRC Population Health Scientist postdoctoral award (grant no. G0902118).

## Figures and Tables

**Table 1:** Precision input value for structure with evidence available for all contrasts.

**(a) Evidence scenario and description**		**(b) Precision (variance) input by level of evidence**			**(c)**${\hat{V}}_{\mathrm{AB}}$**obtained for an IC using values in (b)**		
**Scenario**	**Description**	**Direct A vs. B (**Fig. 1**A)**	**All first-order indirect (**Fig. 1**D)**	**All second-order indirect (**Fig. 1**F)**	**Direct evidence**	**First-order IC**	**Second-order IC**
1	Evidence available in equal amounts for all comparisons	*P* = 1 (*V* = 1)	*P* = 1 (*V* = 1)	*P* = 1 (*V* = 1)	1	2	3
2	Sparse network; a few small trials/weak evidence available for direct and first order. Stronger evidence for second order	*P* = 0.5 (*V* = 2)	*P* = 0.75 (*V* = 1.33)	*P* = 1 (*V* = 1)	2	2.66	3.66
3	Few trials/weak direct evidence on focal treatments. More evidence for first- and second-order comparisons	*P* = 0.5 (*V* = 2)	*P* = 2 (*V* = 0.5)	*P* = 5 (*V* = 0.2)	2	1	1.2
4	Strong direct evidence available for focal treatments. All other evidence weaker	*P* = 5 (*V* = 0.2)	*P* = 2 (*V* = 0.5)	*P* = 1 (*V* = 1)	0.2	1	2
5	Well-populated network; several trials. Strong evidence available for each comparison	*P* = 5 (*V* = 0.2)	*P* = 3 (*V* = 0.33)	*P* = 3 (*V* = 0.33)	0.2	0.66	0.99

*Notes.* Evidence scenarios and corresponding precision input values for each level of evidence in the six-treatment network. “All first-order indirect” refers to evidence on a treatment contrast that contributes to a first-order IC, i.e., A vs. C, …, B vs. F. The variance ${\overset{⌢}{V}}_{\mathrm{AB}}^{I}$ for a first-order IC is formed, e.g., ${\overset{⌢}{V}}_{\mathrm{AB}}^{I}={\overset{⌢}{V}}_{\mathrm{AC}}^{D}+{\overset{⌢}{V}}_{\mathrm{BC}}^{D}$ “All second-order indirect” refers to evidence on a treatment contrast that contributes to a second-order IC, i.e., C vs. D, …, E vs. F. The variance ${\overset{⌢}{V}}_{\mathrm{AB}}^{I}$ for a second-order IC is formed, e.g., ${\overset{⌢}{V}}_{\mathrm{AB}}^{I}={\overset{⌢}{V}}_{\mathrm{AD}}^{D}+{\overset{⌢}{V}}_{\mathrm{CD}}^{D}+{\overset{⌢}{V}}_{\mathrm{BC}}^{D}$.

IC, indirect comparison; *P*, precision; *V*, variance.

## Footnotes

Supplemental material accompanying this article can be found in or, if a hard copy of article, at http://dx.doi.org/10.1016/j.jval.2015.03.0792 or, if a hard copy of article, at www.valueinhealthjournal.com/issues (select volume, issues, and articles).
