# Supplementary material for: Extending Treatment Networks in Health Technology Assessment: How Far Should We Go?
Source: Value Health. 2015 Jul;18(5):673–81. doi: 10.1016/j.jval.2015.03.1792 (PMC4553939; doi:10.1016/j.jval.2015.03.1792)
Supplement: Supplementary file 1 — Supplementary Material [file mmc1.pdf]

Appendix 1: Computing posterior precision P_{AB}^{NMA}

Let y be a vector of treatment effect estimates (for example log-odds ratios) from the n studies included in the NMA with corresponding empirical covariance matrix Σ_y, which is a diagonal matrix with the study-specific variances (standard errors squared) on the diagonal. We assume a Normal likelihood:

$$y \sim N(\mu, \Sigma_y)$$

where μ is a vector of n study-specific mean treatment effects, that are related to the vector of p basic parameters, $\beta = (\theta_{AB}, \theta_{AC}, \theta_{AD}, \ldots)$, through a design matrix X that describes the consistency relations (equation 1). For example in a 4 treatment network with treatments {A,B,C,D} the row of X corresponding to an AB study would be (1,0,0), for an AD study it would be (0,0,1), for a BC study it would be (-1,1,0), for a CD study it would be (0,-1,1) etc.

For a fixed effect model $\mu = X\beta$, and the standard weighted least-squares regression solution(1) gives:

$$\hat{\beta} \sim N\left(\left(X^T \Sigma^{-1} X\right)^{-1} X^T \Sigma^{-1} y, \left(X^T \Sigma^{-1} X\right)^{-1}\right). \qquad \text{(A1.1)}$$

P_{AB}^{NMA} is the reciprocal of the (1,1)th element of $\left(X^T \Sigma^{-1} X\right)^{-1}$. We note that this does not depend on the observed treatment effect estimates, y.

For a random effects model $\mu \sim N\left(X\beta, \Sigma_\mu\right)$ where Σ_μ is a diagonal matrix with each element of the diagonal equal to the between study variance, τ. We assume that the between study variance τ is known and fixed, and give independent flat Normal priors for the basic parameters β with prior precision tending to 0. We follow Gelman et al (2004)(2) section 15.3 to write the hierarchical random effects model as a single normal regression model by extending the data and parameters as follows. The single normal regression model is:

$$y_* \sim N(X_* \gamma_*, \Sigma_*) \qquad \text{(A1.2)}$$

where we define

$y_* = (y, 0_n, 0_p)$ where 0_n is a vector of n 0's,

$X_* = \begin{bmatrix} \mathrm{I}_{n\times n} & 0_{n\times p} \\ \mathrm{I}_{n\times n} & -X \\ 0_{p\times n} & \mathrm{I}_{p\times p} \end{bmatrix}$ where $0_{n\times p}$ is an $n\times p$ matrix of 0's and $\mathrm{I}_{n\times n}$ an $n\times n$ identity matrix, (A1.3)

$\gamma_* = (\mu, \beta)$, and

Σ_* a diagonal ($(2n+p)\times(2n+p)$ matrix with the diagonal equal to the n study-specific variances, the between study variance repeated n times, and 0 repeated p times. (A1.4)

The weighted least squares solution given in equation (A1.1) can be applied to the model (A1.2) to give the posterior for the extended parameter vector γ_* (also given in the 1st edition of Gelmans book(3):

$$\gamma_* \sim N\left(\left(X_*^T\Sigma_*^{-1}X_*\right)^{-1} X_*^T\Sigma_*^{-1}y_*, \left(X_*^T\Sigma_*^{-1}X_*\right)^{-1}\right). \qquad \text{(A1.5)}$$

Note that we are only interested in the precision of the basic parameters, which is the bottom right $p\times p$ sub-matrix of $\left(X_*^T\Sigma_*^{-1}X_*\right)^{-1}$, and in particular the precision P_{AB}^{NMA} of θ_{AB} is the $(n+1, n+1)th$ element of $\left(X_*^T\Sigma_*^{-1}X_*\right)^{-1}$. Note that this also does not depend on the magnitude of the observed treatment effect estimates, y . We can compute $\left(X_*^T\Sigma_*^{-1}X_*\right)^{-1}$ for a given design matrix X , study-specific empirical variances, Σ_y , and between study variance τ. We computed this using the statistical software R (http://www.r-project.org/). Note that due to the zero elements in the bottom p rows of X_* and Σ_*, $\left(X_*^T\Sigma_*^{-1}X_*\right)^{-1}$ is unchanged if these p rows are omitted from the computation, and we take advantage of this in our R code.

Example R code for the Fixed Effect Model

```
#V is a vector of variances for the observed data
V<-c(1,1,1,1,1)

#Design matrix
ndata<-5                                                # number of data inputs
npars<-3                                                # number of basic parameters (=number of treatments minus 1)
X<- matrix(c(1,0,0,  0,1,0,  -1,1,0,   -1,0,1,  0,-1,1), ndata,npars,byrow=T)       #design matrix where 1,0,0
denotes AB parameter, 0,1,0 AC parameter, -1,1,0 BC parameter, -1,0,1 BD parameter and 0,-1,1 CD parameter.
#Sigma ia a covariance matrix for the observed data, and P the precision matrix (P = inverse of Sigma)
Sigma<-diag(V,ndata,ndata)
P<-diag(1/V,ndata,ndata)

#Compute covariance matrix for the pooled NMA estimates
PX<-P%*%X                                  #Performs matrix multiplication PX
Z<-t(X)%*%PX                               #Performs matrix multiplication XPX
Var<-solve(Z)                              #Finds the inverse of XPX. Var is the covariance matrix given in eq.
(A1.1)
VarAB<-Var[1,1]                            #Variance of AB estimate is the [1,1]th element of Var
VarAB                                      #estimate of variance for the AB parameter
```

Example R code for the Random Effects Model based on Gelman et al (2004)

```
#V is a vector of variances for the observed data
V<-c(1,1,1,1,1)

#tausq is the between studies heterogeneity parameter
tausq<-0.5

#Design matrix
ndata<-5                                   # number of data inputs
npars<-3                                   # number of basic parameters (=number of treatments minus 1)
X<- matrix(c(1,0,0,  0,1,0,  -1,1,0,   -1,0,1,  0,-1,1), ndata,npars,byrow=T)       #design matrix where 1,0,0
denotes AB parameter, 0,1,0 AC parameter, -1,1,0 BC parameter, -1,0,1 BD parameter and 0,-1,1 CD parameter.


#Augmented design matrix to allow for hierarchical structure (A1.3)
X1<-diag(,ndata,ndata)
```

```
X0<-matrix(rep(0,ndata*npars),ndata,npars)
Xstar<-rbind(cbind(X1,X0),cbind(X1,X))

#Augmented precision matrix to allow for hierarchical structure (A1.4)
Vstar<-c(V,rep(tausq,ndata))
Pstar<-diag(1/Vstar,2*ndata,2*ndata)

#Compute covariance matrix for the pooled NMA estimates (A1.5)
XP<-Pstar%*%Xstar             #Performs matrix multiplication PX
Z<-t(Xstar)%*%XP                    #Performs matrix multiplication XPX
Var<-solve(Z)                       #Finds the inverse of XPX. Var is the covariance matrix given in equation (A1.4)
VarAB<-Var[(ndata+1),(ndata+1)]     #Variance of AB estimate is the [ndata+1,ndata+1]th element of Var
VarAB                               #estimate of variance for the AB parameter
```

References

1. Hedges LV, Olkin I. Statistical Methods for Meta-analysis. London: Academic Press; 1985.
2. Gelman A, Carlin JB, Stern HS, Rubin DB. Bayesian Data Analysis. 2nd ed: Chapman & Hall; 2004.
3. Gelman A, Carlin JG, Stern HS, Rubin DB. Bayesian data analysis. 1st ed. London: Chapman and Hall; 1995.

Appendix 1: supplementary tables

		Variance assumed under each scenario				
		1 trial per comparison (1)	*AB weakest link, IC trials weaker (2)*	*AB weakest link, IC trials strong (3)*	*AB strongest link, IC trials weaker (4)*	*AB strongest link, IC trials strong (5)*
**Network Comparisons**	AB	1	2	2	0.2	0.2
	AC to BF	1	1.33	0.5	0.5	0.33
	CD to EF	1	1	0.2	1	0.33

Table 1: Explanation of variance assumed under each of the five scenarios considered.

Star network: In tables 2 to 5 the following scenarios are reported:
Scenario 1: “1 trial per comparison”: Equal variance across the network. Each comparison XY represents 1 meta-analysis with variance equal to 1.
Scenario 2: “AB weakest link, IC trials weaker” AB comparison is the ‘weakest’ link, with the comparisons forming indirect comparisons being weaker.
Scenario 3: “AB weakest link, IC trials strong” AB comparison is the ‘weakest’ link, with the comparisons forming indirect comparisons being stronger.
Scenario 4: “AB strongest link, IC trials weaker”: AB comparison is the ‘strongest’ link, with the comparisons forming indirect comparisons being weaker.
Scenario 5: “AB strongest link, IC trials strong” AB comparison is the ‘strongest’ link, with the comparisons forming indirect comparisons also being strong.

Fig.	Direct evidence available	1 trial per comparison (1)			AB weakest link, IC trials weaker (2)			AB weakest link, IC trials strong (3)			AB strongest link, IC trials weaker (4)			AB strongest link, IC trials strong (5)		
		Std MA	NMA	Increase %	Std MA	NMA	Increase %	Std MA	NMA	Increase %	Std MA	NMA	Increase %	Std MA	NMA	Increase %
1b	AB AC AD AE AF	1.00	1.00	0	0.50	0.50	0	0.50	0.50	0	5.00	5.00	0	5.00	5.00	0
1c	+ BC	1.00	1.50	50	0.50	0.88	75	0.50	1.50	200	5.00	6.00	20	5.00	6.50	30
1d	+ BC BD	1.00	2.00	100	0.50	1.25	150	0.50	2.50	400	5.00	7.00	40	5.00	8.00	60
1e	+ BC BD BE	1.00	2.50	150	0.50	1.63	225	0.50	3.50	600	5.00	8.00	60	5.00	9.50	90
1e	+ BC BD BE BF	1.00	3.00	200	0.50	2.00	300	0.50	4.50	800	5.00	9.00	80	5.00	11.01	120
-	+ BC BD BE BF CD	1.00	3.00	200	0.50	2.00	300	0.50	4.50	800	5.00	9.00	80	5.00	11.01	120
-	+ BC BD BE BF CD CE	1.00	3.00	200	0.50	2.00	300	0.50	4.50	800	5.00	9.00	80	5.00	11.01	120
-	+ BC BD BE BF CD CE CF	1.00	3.00	200	0.50	2.00	300	0.50	4.50	800	5.00	9.00	80	5.00	11.01	120
-	+ BC BD BE BF CD CE CF DE	1.00	3.00	200	0.50	2.00	300	0.50	4.50	800	5.00	9.00	80	5.00	11.01	120
-	+ BC BD BE BF CD CE CF DE DF	1.00	3.00	200	0.50	2.00	300	0.50	4.50	800	5.00	9.00	80	5.00	11.01	120
1f	Entire network connected	1.00	3.00	200	0.50	2.00	300	0.50	4.50	800	5.00	9.00	80	5.00	11.01	120

Table 2 Fixed effect analysis for a star network with 6 treatments. Comparison of precision gained for treatment contrast A vs B from expanding network of treatments under five separate scenarios with that achieved from a standard, pairwise meta-analysis. Columns report the precision of A vs B treatment effect estimate (log odds scale) from a pairwise meta-analysis of A vs B (std MA), the precision of A vs B from a network meta-analysis (NMA) and the percentage difference between NMA and std MA (Increase %). Each increasing level of the network is illustrated with reference to Figure 1 in main paper. Explanation of variances assumed for each comparison given in Table 1 above.

Figure	Direct evidence available	1 trial per comparison (1)			AB weakest link, IC trials weaker (2)			AB weakest link, IC trials strong (3)			AB strongest link, IC trials weaker (4)			AB strongest link, IC trials strong (5)		
		Std MA	NMA	% Increase	Std MA	NMA	% Increase	Std MA	NMA	% Increase	Std MA	NMA	% Increase	Std MA	NMA	Increase
1b	AB AC AD AE AF	0.91	0.91	0	0.48	0.48	0	0.48	0.48	0	3.33	3.33	0	3.33	3.33	0
1c	+ BC	0.91	1.36	50	0.48	0.83	73	0.48	1.31	175	3.33	4.17	25	3.33	4.49	35
1e	+ BC BD	0.91	1.82	100	0.48	1.17	147	0.48	2.14	350	3.33	5	50	3.33	5.64	69
1e	+ BC BD BE	0.91	2.27	150	0.48	1.52	220	0.48	2.98	525	3.33	5.83	75	3.33	6.8	104
1e	+ BC BD BE BF	0.91	2.73	200	0.48	1.87	293	0.48	3.81	700	3.33	6.67	100	3.33	7.95	139
-	+ BC BD BE BF CD	0.91	2.73	200	0.48	1.87	293	0.48	3.81	700	3.33	6.67	100	3.33	7.95	139
-	+ BC BD BE BF CD CE	0.91	2.73	200	0.48	1.87	293	0.48	3.81	700	3.33	6.67	100	3.33	7.95	139
-	+ BC BD BE BF CD CE CF	0.91	2.73	200	0.48	1.87	293	0.48	3.81	700	3.33	6.67	100	3.33	7.95	139
-	+ BC BD BE BF CD CE CF DE	0.91	2.73	200	0.48	1.87	293	0.48	3.81	700	3.33	6.67	100	3.33	7.95	139
-	+ BC BD BE BF CD CE CF DE DF	0.91	2.73	200	0.48	1.87	293	0.48	3.81	700	3.33	6.67	100	3.33	7.95	139
1f	Entire network connected	0.91	2.73	200	0.48	1.87	293	0.48	3.81	700	3.33	6.67	100	3.33	7.95	139

Table 3: Random effects analysis for a star network with 6 treatments. Comparison of precision gained for treatment contrast A vs B from expanding network of treatments under five separate scenarios with that achieved from a standard, pairwise meta-analysis. **Assuming 'low' between trial heterogeneity (τ^2 = 0.1).** Columns report the precision of A vs B treatment effect estimate from a pairwise meta-analysis of A vs B (std MA), the precision of A vs B from a network meta-analysis (NMA) and the percentage difference between NMA and std MA (Increase %). Each increasing level of the network is illustrated with reference to Figure 1 in main paper. Explanation of variances assumed for each comparison given in Table 1 above.

Figure	Direct evidence available	1 trial per comparison (1)			AB weakest link, IC trials weaker (2)			AB weakest link, IC trials strong (3)			AB strongest link, IC trials weaker (4)			AB strongest link, IC trials strong (5)		
		Std MA	NMA	% Increase	Std MA	NMA	% Increase	Std MA	NMA	% Increase	Std MA	NMA	% Increase	Std MA	NMA	% Increase
1b	AB AC AD AE AF	0.67	0.67	0	0.40	0.40	0	0.40	0.40	0	1.43	1.43	0	1.43	1.43	0
1c	+ BC	0.67	1.00	50	0.40	0.67	68	0.40	0.90	125	1.43	1.93	35	1.43	2.03	42
1e	+ BC BD	0.67	1.33	100	0.40	0.95	136	0.40	1.40	250	1.43	2.43	70	1.43	2.63	84
1e	+ BC BD BE	0.67	1.67	150	0.40	1.22	205	0.40	1.90	375	1.43	2.93	105	1.43	3.23	126
1e	+ BC BD BE BF	0.67	2.00	200	0.40	1.49	273	0.40	2.40	500	1.43	3.43	140	1.43	3.83	168
-	+ BC BD BE BF CD	0.67	2.00	200	0.40	1.49	273	0.40	2.40	500	1.43	3.43	140	1.43	3.83	168
-	+ BC BD BE BF CD CE	0.67	2.00	200	0.40	1.49	273	0.40	2.40	500	1.43	3.43	140	1.43	3.83	168
-	+ BC BD BE BF CD CE CF	0.67	2.00	200	0.40	1.49	273	0.40	2.40	500	1.43	3.43	140	1.43	3.83	168
-	+ BC BD BE BF CD CE CF DE	0.67	2.00	200	0.40	1.49	273	0.40	2.40	500	1.43	3.43	140	1.43	3.83	168
-	+ BC BD BE BF CD CE CF DE DF	0.67	2.00	200	0.40	1.49	273	0.40	2.40	500	1.43	3.43	140	1.43	3.83	168
1f	Entire network connected	0.67	2.00	200	0.40	1.49	273	0.40	2.40	500	1.43	3.43	140	1.43	3.83	168

Table 4: Random effects analysis for a star network with 6 treatments. Comparison of precision gained for treatment contrast A vs B from expanding network of treatments under five separate scenarios with that achieved from a standard, pairwise meta-analysis. Assuming 'medium' between trial heterogeneity ($\tau^2 = 0.5$). Columns report the precision of A vs B treatment effect estimate from a pairwise meta-analysis of A vs B (std MA), the precision of A vs B from a network meta-analysis (NMA) and the percentage difference between NMA and std MA (Increase %). Each increasing level of the network is illustrated with reference to Figure 1 in main paper. Explanation of variances assumed for each comparison given in Table 1 above.

Fig	Direct evidence available	1 trial per comparison (1)			AB weakest link, IC trials weaker (2)			AB weakest link, IC trials strong (3)			AB strongest link, IC trials weaker (4)			AB strongest link, IC trials strong (5)		
		Std MA	NMA	% Increase	Std MA	NMA	% Increase	Std MA	NMA	% Increase	Std MA	NMA	% Increase	Std MA	NMA	% Increase
1b	AB AC AD AE AF	0.50	0.5	0	0.33	0.33	0	0.33	0.33	0	0.83	0.83	0	0.83	0.83	0
1c	+ BC	0.50	0.75	50	0.33	0.55	66	0.33	0.67	102	0.83	1.17	41	0.83	1.21	46
1e	+ BC BD	0.50	1.00	100	0.33	0.76	131	0.33	1.00	203	0.83	1.50	81	0.83	1.58	91
1e	+ BC BD BE	0.50	1.25	150	0.33	0.98	196	0.33	1.33	304	0.83	1.83	121	0.83	1.96	136
1e	+ BC BD BE BF	0.50	1.50	200	0.33	1.19	261	0.33	1.67	405	0.83	2.17	161	0.83	2.33	181
-	+ BC BD BE BF CD	0.50	1.50	200	0.33	1.19	261	0.33	1.67	405	0.83	2.17	161	0.83	2.33	181
-	+ BC BD BE BF CD CE	0.50	1.50	200	0.33	1.19	261	0.33	1.67	405	0.83	2.17	161	0.83	2.33	181
-	+ BC BD BE BF CD CE CF	0.50	1.50	200	0.33	1.19	261	0.33	1.67	405	0.83	2.17	161	0.83	2.33	181
-	+ BC BD BE BF CD CE CF DE	0.50	1.50	200	0.33	1.19	261	0.33	1.67	405	0.83	2.17	161	0.83	2.33	181
-	+ BC BD BE BF CD CE CF DE DF	0.50	1.50	200	0.33	1.19	261	0.33	1.67	405	0.83	2.17	161	0.83	2.33	181
1f	Entire network connected	0.50	1.50	200	0.33	1.19	261	0.33	1.67	405	0.83	2.17	161	0.83	2.33	181

Table 5: Random effects analysis for a star network with 6 treatments. Comparison of precision gained for treatment contrast A vs B from expanding network of treatments under five separate scenarios with that achieved from a standard, pairwise meta-analysis. Assuming 'high' between trial heterogeneity (τ^2 = 1). Columns report the precision of A vs B treatment effect estimate from a pairwise meta-analysis of A vs B (std MA), the precision of A vs B from a network meta-analysis (NMA) and the percentage difference between NMA and std MA (Increase %). Each increasing level of the network is illustrated with reference to Figure 1. Explanation of variances assumed for each comparison given in Table 1 above.

Tables 6 to 9: Absence of first-order indirect comparisons. The following scenarios are reported:

Scenario 1: "1 trial per comparison": Equal variance across the network. Each comparison XY represents 1 meta-analysis with variance equal to 1.
Scenario 2: "**1st order IC trials weak, 2nd order slightly stronger**": comparisons forming 1st order indirect comparisons weak, 2nd order slightly stronger but still weak.
Scenario 3: "**1st order IC trials strong, 2nd order strong**": comparisons forming 1st order indirect comparisons is strong but 2nd order IC stronger still.
Scenario 4: "**1st order IC trials strong, 2nd order weaker**": comparisons forming indirect comparisons strong 2nd order being weaker.
Scenario 5: "**1st order IC trials strong, 2nd order strong**" comparisons forming indirect comparisons strong and 2nd order IC also being strong.

Fig.	Direct evidence available	1 trial per comparison (1)			AB weakest link 1st order IC trials weak, 2nd order weak (2)			AB weakest link 1st order IC trials strong, 2nd order strong (3)			AB strongest link 1st order IC trials strong, 2nd order weaker (4)			AB strongest link 1st order IC trials strong, 2nd order strong (5)		
		Std MA	NMA	Increase %	Std MA	NMA	Increase %	Std MA	NMA	Increase %	Std MA	NMA	Increase %	Std MA	NMA	Increase %
2a	AB (AD AE BC BF)	1.00	1.00	0	0.50	0.50	0	0.50	0.50	0	5.00	5.00	0	5.00	5.00	0
-	1 2nd order loop	1.00	1.33	33	0.50	0.77	54	0.50	1.33	166	5.00	5.50	10	5.00	6.01	20
2b	1 1st order & 1 2nd order loop	1.00	1.83	83	0.50	0.93	86	0.50	1.93	286	5.00	5.66	13	5.00	6.52	30
2c	2 2nd-order loops (2a CD EF)	1.00	1.67	67	0.50	1.05	110	0.50	2.17	334	5.00	6.00	20	5.00	7.02	40
2d	2 1st order loops (2a AC AF)	1.00	2.00	100	0.50	1.25	150	0.50	2.50	400	5.00	7.00	40	5.00	8.03	61
2e	2 1st & 2 2nd order loops (2c & 2d)	1.00	2.20	120	0.50	1.42	184	0.50	3.03	506	5.00	7.28	46	5.00	8.64	73
2f	4 1st & 2 2nd order loops (2e BD BE)	1.00	3.00	200	0.50	2.00	300	0.50	4.50	800	5.00	9.00	80	5.00	11.06	121
2g	4 1st order loops (2a AC AF BD BE)	1.00	3.00	200	0.50	2.00	300	0.50	4.50	800	5.00	9.00	80	5.00	11.06	121
2h	Fully connected network	1.00	3.00	200	0.50	2.00	300	0.50	4.50	800	5.00	9.00	80	5.00	11.06	121

Table 6 Fixed effect analysis for investigating absence of 1st order indirect evidence. Comparison of precision gained for treatment contrast A vs B under five separate scenarios with that achieved from a standard, pairwise meta-analysis. Columns report the precision of A vs B treatment effect estimate from a pairwise meta-analysis of A vs B (std MA), the precision of A vs B from a network meta-analysis (NMA) and the percentage difference between NMA and std MA (Increase %). Each increasing level of the network is illustrated with reference to Figure 2. Explanation of variances assumed for each comparison given in Table 1 in main paper.

Fig.	Direct evidence available	1 trial per comparison (1)			AB weakest link 1st order IC trials weak, 2nd order weak (2)			AB weakest link 1st order IC trials strong, 2nd order strong (3)			AB strongest link 1st order IC trials strong, 2nd order weaker (4)			AB strongest link 1st order IC trials strong, 2nd order strong (5)		
		Std MA	NMA	Increase %	Std MA	NMA	Increase %	Std MA	NMA	Increase %	Std MA	NMA	Increase %	Std MA	NMA	Increase %
2a	AB (AD AE BC BF)	0.91	0.91	0	0.48	0.48	0	0.48	0.48	0	3.33	3.33	0	3.33	3.33	0
-	1 2nd order loop	0.91	1.21	33	0.48	0.73	52	0.48	1.14	138	3.33	3.77	13	3.33	4.11	23
2b	1 1st order & 1 2nd order loop	0.91	1.66	82	0.48	1.08	125	0.48	1.98	313	3.33	4.60	38	3.33	5.27	58
2c	2 2nd-order loops (2a CD EF)	0.91	1.52	67	0.48	0.98	104	0.48	1.81	277	3.33	4.20	26	3.33	4.88	47
2d	2 1st order loops (2a AC AF)	0.91	1.82	100	0.48	1.18	146	0.48	2.14	346	3.33	5.00	50	3.33	5.66	70
2e	2 1st & 2 2nd order loops (2d & 2c)	0.91	2.00	120	0.48	1.33	177	0.48	2.56	433	3.33	5.25	58	3.33	6.12	84
2f	4 1st & 2 2nd order loops (2e BD BE)	0.91	2.73	200	0.48	1.87	290	0.48	3.81	694	3.33	6.66	100	3.33	7.98	140
2g	4 1st order loops (2a AC AF BD BE)	0.91	2.73	200	0.48	1.87	290	0.48	3.81	694	3.33	6.66	100	3.33	7.98	140
2h	Fully connected network	0.91	2.73	200	0.48	1.87	290	0.48	3.81	694	3.33	6.66	100	3.33	7.98	140

Table 7: Random effects analysis for investigating absence of 1st order indirect evidence. Comparison of precision gained for treatment contrast A vs B under five separate scenarios with that achieved from a standard, pairwise meta-analysis. **Assuming 'low' between trial heterogeneity (τ^2 = 0.1).** Columns report the precision of A vs B treatment effect estimate from a pairwise meta-analysis of A vs B (std MA), the precision of A vs B from a network meta-analysis (NMA) and the percentage difference between NMA and std MA (Increase %). Each increasing level of the network is illustrated with reference to Figure 2. Explanation of variances assumed for each comparison given in Table 1 in main paper.

Fig.	Direct evidence available	1 trial per comparison (1)			AB weakest link 1st order IC trials weak, 2nd order weak (2)			AB weakest link 1st order IC trials strong, 2nd order strong (3)			AB strongest link 1st order IC trials strong, 2nd order weaker (4)			AB strongest link 1st order IC trials strong, 2nd order strong (5)		
		Std MA	NMA	Increase %	Std MA	NMA	Increase %	Std MA	NMA	Increase %	Std MA	NMA	Increase %	Std MA	NMA	Increase %
2a	AB (AD AE BC BF)	0.66	0.66	0	0.40	0.40	0	0.40	0.40	0	1.43	1.43	0	1.43	1.43	0
-	1 2nd order loop	0.66	0.89	35	0.40	0.59	48	0.40	0.77	93	1.43	1.71	20	1.43	1.83	28
2b	1 1st order & 1 2nd order loop	0.66	1.22	85	0.40	0.87	118	0.40	1.27	218	1.43	2.21	55	1.43	2.42	69
2c	2 2nd-order loops (2a CD EF)	0.66	1.11	68	0.40	0.79	98	0.40	1.14	185	1.43	2.00	40	1.43	2.23	56
2d	2 1st order loops (2a AC AF)	0.66	1.33	102	0.40	0.95	138	0.40	1.40	250	1.43	2.43	70	1.43	2.63	84
2e	2 1st & 2 2nd order loops (2d & 2c)	0.66	1.47	123	0.40	1.06	165	0.40	1.63	308	1.43	2.60	82	1.43	2.87	101
2f	4 1st & 2 2nd order loops (2e BD BE)	0.66	2.00	203	0.40	1.49	273	0.40	2.40	500	1.43	3.43	140	1.43	3.84	169
2g	4 1st order loops (2a AC AF BD BE)	0.66	2.00	203	0.40	1.49	273	0.40	2.40	500	1.43	3.43	140	1.43	3.84	169
2h	Fully connected network	0.66	2.00	203	0.40	1.49	273	0.40	2.40	500	1.43	3.43	140	1.43	3.84	169

Table 8: Random effects analysis for investigating absence of 1st order indirect evidence. Comparison of precision gained for treatment contrast A vs B under five separate scenarios with that achieved from a standard, pairwise meta-analysis. **Assuming 'medium' between trial heterogeneity (τ^2 = 0.5).**Columns report the precision of A vs B treatment effect estimate from a pairwise meta-analysis of A vs B (std MA), the precision of A vs B from a network meta-analysis (NMA) and the percentage difference between NMA and std MA (Increase %). Each increasing level of the network is illustrated with reference to Figure 2. Explanation of variances assumed for each comparison given in main paper.

Fig.	Direct evidence available	1 trial per comparison (1)			AB weakest link 1st order IC trials weak, 2nd order weak (2)			AB weakest link 1st order IC trials strong, 2nd order strong (3)			AB strongest link 1st order IC trials strong, 2nd order weaker (4)			AB strongest link 1st order IC trials strong, 2nd order strong (5)		
		Std MA	NMA	Increase %	Std MA	NMA	Increase %	Std MA	NMA	Increas e %	Std MA	NMA	Increas e %	Std MA	NMA	Increase %
2a	AB (AD AE BC BF)	0.50	0.50	0	0.33	0.33	0	0.33	0.33	0	0.83	0.83	0	0.83	0.83	0
-	1 2nd order loop	0.50	0.66	32	0.33	0.48	45	0.33	0.57	73	0.83	1.03	24	0.83	1.08	30
2b	1 1st order & 1 2nd order loop	0.50	0.92	84	0.33	0.70	112	0.33	0.90	173	0.83	1.37	65	0.83	1.46	76
2c	2 2nd-order loops (2a CD EF)	0.50	0.83	66	0.33	0.63	91	0.33	0.81	145	0.83	1.23	48	0.83	1.33	60
2d	2 1st order loops (2a AC AF)	0.50	1.00	100	0.33	0.76	130	0.33	1.00	203	0.83	1.50	81	0.83	1.59	92
2e	2 1st & 2 2nd order loops (2d & 2c)	0.50	1.10	120	0.33	0.85	158	0.33	1.15	248	0.83	1.62	95	0.83	1.74	110
2f	4 1st & 2 2nd order loops (2e BD BE)	0.50	1.50	200	0.33	1.19	261	0.33	1.66	403	0.83	2.17	161	0.83	2.34	182
2g	4 1st order loops (2a AC AF BD BE)	0.50	1.50	200	0.33	1.19	261	0.33	1.66	403	0.83	2.17	161	0.83	2.34	182
2h	Fully connected network	0.50	1.50	200	0.33	1.19	261	0.33	1.66	403	0.83	2.17	161	0.83	2.34	182

Table 9: Random effects analysis for investigating absence of 1st order indirect evidence. Comparison of precision gained for treatment contrast A vs B under five separate scenarios with that achieved from a standard, pairwise meta-analysis. **Assuming 'high' between trial heterogeneity (τ^2 = 1).** Columns report the precision of A vs B treatment effect estimate from a pairwise meta-analysis of A vs B (std MA), the precision of A vs B from a network meta-analysis (NMA) and the percentage difference between NMA and std MA (Increase %). Each increasing level of the network is illustrated with reference to Figure 2. Explanation of variances assumed for each comparison given in main paper.
