# Supplementary material for: Extending Treatment Networks in Health Technology Assessment: How Far Should We Go?
Source: Value Health. 2015 Jul;18(5):673–81. doi: 10.1016/j.jval.2015.03.1792 (PMC4553939; doi:10.1016/j.jval.2015.03.1792)
Supplement: Supplementary file 2 — Supplementary Material [file mmc2.pdf]

## Appendix 2: sensitivity analysis for 6 treatment network for all available evidence

| Level of network connectedness | Scenarios and evidence structures | | | | | | | | | | | |
|---|---|---|---|---|---|---|---|---|---|---|---|---|
| | (1) Direct weak, IC weak | | | (2) Direct weak, IC strong | | | (3) Direct strong, IC weak | | | (4) Direct strong, IC strong | | |
| | a | b | c | a | b | c | a | b | c | a | b | c |
| AB AC AD AE AF | 0.25 | 0.05 | 0.05 | 0.25 | 0.05 | 0.05 | 2.5 | 0.5 | 0.5 | 2.5 | 0.5 | 0.5 |
| + BC | 0.44 | 0.09 | 0.09 | 0.75 | 0.15 | 0.14 | 3 | 0.6 | 0.59 | 3.25 | 0.65 | 0.64 |
| + BC BD | 0.63 | 0.13 | 0.13 | 1.25 | 0.25 | 0.21 | 3.5 | 0.7 | 0.68 | 4 | 0.8 | 0.83 |
| + BC BD BE | 0.81 | 0.16 | 0.16 | 1.75 | 0.35 | 0.28 | 4 | 0.8 | 0.75 | 4.75 | 0.95 | 0.99 |
| + BC BD BE BF | 1 | 0.2 | 0.19 | 2.25 | 0.45 | 0.37 | 4.5 | 0.9 | 0.85 | 5.5 | 1.1 | 1.14 |
| + BC BD BE BF CD | 1 | 0.2 | 0.19 | 2.25 | 0.45 | 0.37 | 4.5 | 0.9 | 0.85 | 5.5 | 1.1 | 1.14 |
| + BC BD BE BF CD CE | 1 | 0.2 | 0.19 | 2.25 | 0.45 | 0.37 | 4.5 | 0.9 | 0.85 | 5.5 | 1.1 | 1.14 |
| + BC BD BE BF CD CE CF | 1 | 0.2 | 0.19 | 2.25 | 0.45 | 0.37 | 4.5 | 0.9 | 0.85 | 5.5 | 1.1 | 1.14 |
| + BC BD BE BF CD CE CF DE | 1 | 0.2 | 0.19 | 2.25 | 0.45 | 0.37 | 4.5 | 0.9 | 0.85 | 5.5 | 1.1 | 1.14 |
| + BC BD BE BF CD CE CF DE DF | 1 | 0.2 | 0.19 | 2.25 | 0.45 | 0.37 | 4.5 | 0.9 | 0.85 | 5.5 | 1.1 | 1.14 |
| + BC BD BE BF CD CE CF DE DF EF | 1 | 0.2 | 0.19 | 2.25 | 0.45 | 0.37 | 4.5 | 0.9 | 0.85 | 5.5 | 1.1 | 1.14 |

Results of sensitivity analysis for fixed effect model to evaluate impact of increasing the variance input values and also allowing them to vary within evidence level. Scenarios are described as:

**Scenario 1:** “AB weakest link, IC trials weaker” AB comparison is the ‘weakest’ link, with the comparisons forming indirect comparisons being weaker (less precise).
**Scenario 2**: “AB weakest link, IC trials strong” AB comparison is the ‘weakest’ link, with the comparisons forming indirect comparisons being stronger (more precise).
**Scenario 3:** “AB strongest link, IC trials weaker”: AB comparison is the ‘strongest’ link, with the comparisons forming indirect comparisons being weaker (less precise).
**Scenario 4:** “AB strongest link, IC trials strong” AB comparison is the ‘strongest’ link, with the comparisons forming indirect comparisons also being strong (more precise).

Within each scenario variance input values were varied in three ways:

(a) Doubling of values such that for scenario 1: $P^D_{AB} = 0.25; P^D_{AC}$ … $P^D_{BF} = 2.67$ and $P^D_{CD}$ … $P^D_{EF} = 2$
(b) Values increased ten-fold, such that for scenario 1: $P^D_{AB} = 0.05; P^D_{AC}$ … $P^D_{BF} = 0.38$ and $P^D_{CD}$ … $P^D_{EF} = 0.1$
(c) Values arbitrarily varied within level, such that for scenario 1: e.g. $P^D_{AB} = 0.05; P^D_{AC} = 0.075$, $P^D_{AD} = 0.07, P^D_{AE} = 0.08$, $P^D_{AF} = 0.0612$, $P^D_{BC} = 0.08$, $P^D_{BD} = 0.08$, $P^D_{BE} = 0.055, P^D_{BF} = 0.07$ and so on.
