# Supplementary material for: Extending Treatment Networks in Health Technology Assessment: How Far Should We Go?
Source: Value Health. 2015 Jul;18(5):673–81. doi: 10.1016/j.jval.2015.03.1792 (PMC4553939; doi:10.1016/j.jval.2015.03.1792)
Supplement: Supplementary file 3 — Supplementary Material [file mmc3.pdf]

Appendix 3: Sensitivity analysis for impact of second order indirect evidence

Fixed effect indirect missing analysis. Weak direct evidence for AB, weak first order indirect and very strong 2nd-order indirect evidence

Note values of precision of evidence are arbitrary and were chosen for convenience. Precision for each order of evidence reported above results

		**Scenario 1**<br>Direct evidence= 0.2<br>1st order = 0.1<br>2nd order = 20			**Scenario 2**<br>Direct evidence= 0.2<br>1st order = 0.2<br>2nd order = 20			**Scenario 3**<br>Direct evidence= 0.2<br>1st order = 0.5<br>2nd order = 20			**Scenario 4**<br>Direct evidence= 0.2<br>1st order = 0.05<br>2nd order = 20		
**Fig.**	**Evidence included in network**	**Precision gained (% increase)**			**Precision gained (% increase)**			**Precision gained (% increase)**			**Precision gained (% increase)**		
		Std MA	NMA	Increase %	Std MA	NMA	Increase %	Std MA	NMA	Increase %	Std MA	NMA	Increase %
2a	AB (AD AE BC BF)	0.20	0.20	0	0.20	0.20	0	0.20	0.20	0	0.20	0.20	0
-	1 2nd order loop	0.20	0.23	15	0.20	0.27	35	0.20	0.37	85	0.20	0.22	10
-	1 1st order loop	0.20	0.25	25	0.20	0.30	50	0.20	0.45	125	0.20	0.23	15
2b	1 1st order & 1 2nd order loop	0.20	0.30	50	0.20	0.40	100	0.20	0.70	250	0.20	0.25	25
2c	2 2nd-order loops (2a CD EF)	0.20	0.30	50	0.20	0.40	100	0.20	0.69	245	0.20	0.25	25
2d	2 1st order loops (2a AC AF)	0.20	0.30	50	0.20	0.40	100	0.20	0.70	250	0.20	0.25	25
2e	2 1st & 2 2nd order loops (2c & 2d)	0.20	0.33	65	0.20	0.47	135	0.20	0.86	330	0.20	0.27	35
2f	4 1st & 2 2nd order loops (2e BD BE)	0.20	0.40	100	0.20	0.60	200	0.20	1.20	500	0.20	0.30	50
2g	4 1st order loops (2a AC AF BD BE)	0.20	0.40	100	0.20	0.60	200	0.20	1.20	500	0.20	0.30	50
2h	Fully connected network	0.20	0.40	100	0.20	0.60	200	0.20	1.20	500	0.20	0.30	50
